# Effective mitigation of blood culture bottle shortage with diagnostic stewardship interventions

**DOI:** 10.1128/jcm.01701-24

**Published:** 2025-01-24

**Authors:** Jessica Hudson, Guillermo Rodriguez Nava, Mindy Marie Sampson, Amy Chang, Alex Maurice Dussaq, Jorge Luis Salinas, Angela Serbest, Tho Pham, Niaz Banaei

**Affiliations:** 1 Department of Pathology, Stanford University School of Medicine 10624 , Stanford, California, USA; 2 Division of Infectious Diseases & Geographic Medicine, Stanford University School of Medicine 10624 , Stanford, California, USA; 3 Clinical Microbiology Laboratory, Stanford University Department of Pathology 158566 , Stanford, California, USA; Endeavor Health, Evanston, Illinois, USA

**Keywords:** blood culture, stewardship, intervention, shortage

## LETTER

Blood culture serves a critical role in diagnosis and management of bloodstream infections (BSI). In June 2024, the manufacturer of BD BACTEC FX Blood Culture System announced a major shortage of blood culture bottles with a projected supply reduction by 50%. Best practices for blood culture include limiting blood cultures to patients with signs and symptoms consistent with BSI, culturing an optimal blood volume at baseline, and limiting repeat blood cultures (1, 2). Shortly after the shortage started, Stanford Health Care (SHC) implemented two interventions to mitigate the shortage.

A before and after interventional study was conducted at SHC serving adult patients. For each blood culture order, 20 mL of blood was drawn and dispensed into two bottles. Two blood culture orders (three aerobic bottles and one anaerobic bottle) were placed at baseline per institutional protocol. Two interventions were concurrently implemented on 9 July 2024. First, an alert was implemented during blood culture electronic order entry in EPIC to inform providers about the blood culture bottle shortage and the need for judicious utilization of blood cultures in addition to a concise educational table of clinical scenarios for appropriate and inappropriate ordering of blood cultures (Fig. S1). The second intervention used a computer-assisted, order entry algorithm to block repeat blood cultures within 72 hours of baseline cultures (Fig. S2) with links to supporting literature for the rationale of practice. The providers were given an exception criterion to the “Hard Stop” and allowed to repeat blood cultures. The study endpoints were (1) blood culture order volumes, (2) the culture positivity rate, and (3) the rate of repeat blood cultures in 72 hours. These endpoints were compared for 26 weeks before and 15 weeks post-intervention. The Mann-Whitney U test was used to compare unpaired results.

**Fig 1:**
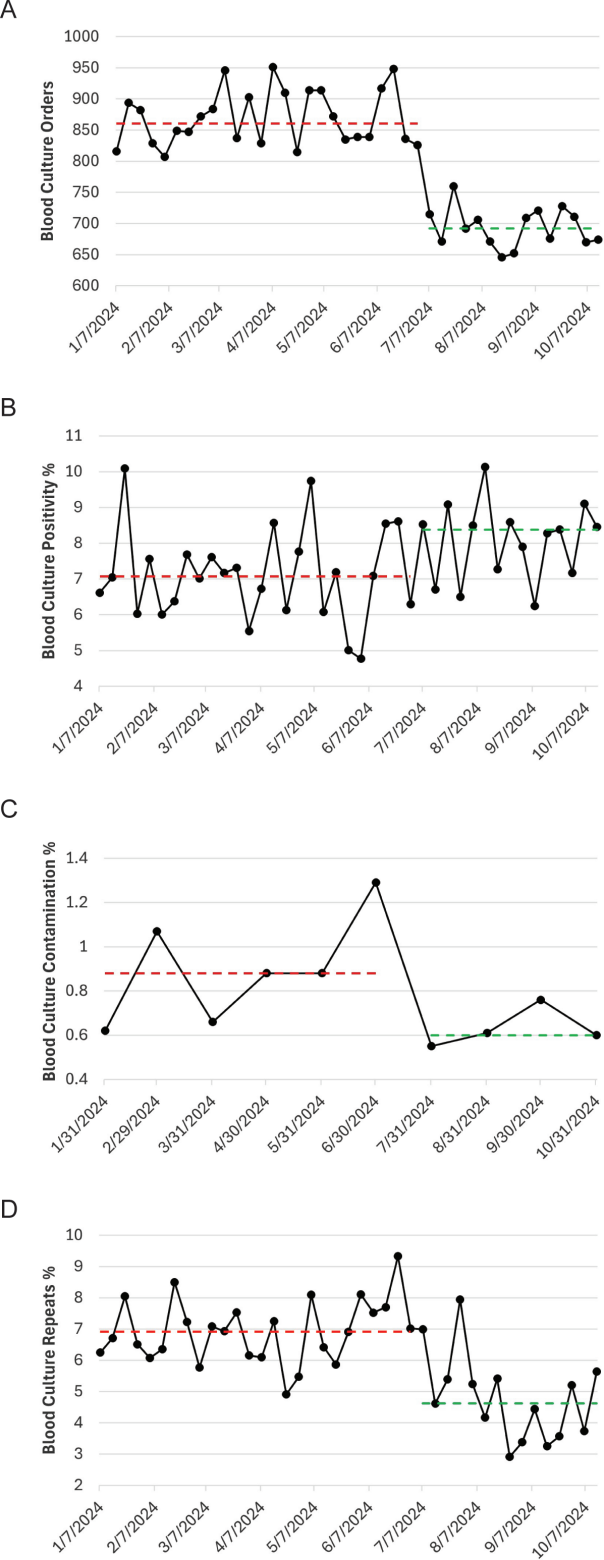
Blood culture utilization, positivity rate, and repeat orders pre- and post-intervention. (**A**) Weekly blood culture order numbers. (**B**) Weekly blood culture positivity rate. (**C**) Monthly blood culture contamination rate. (**D**) Weekly blood culture repeats within 72 hours of a baseline blood culture. Horizontal red and green dash lines show medians for pre-intervention and intervention periods, respectively.

Compared with the pre-interventional period, the median weekly blood culture orders decreased by 19.6% during the interventional period from 861 to 692 orders per week (*P* < 0.001) (Fig. 1A). The median weekly positivity rates were 7.1% and 8.4% (*P* = 0.02) during the pre-intervention and intervention periods, respectively (Fig. 1B). The monthly contamination rate decreased from a median of 1.1% during the pre-intervention period to 0.9% during the intervention period (*P* = 0.52) (Fig. 1C). The weekly percentage of repeat blood cultures within 72 hours declined by 33.2% from a median of 6.9% to 4.6% repeats per week (*P* < 0.001) (Fig. 1D). No adverse events related to blood culture orders were reported to the hospital quality department during the interventional period.

We showed a significant reduction in blood culture utilization and repeat testing and a significant increase in blood culture positivity rate in response to an educational alert and a hard stop in EPIC, respectively. The underlying reasons for rapid provider buy-in are multifactorial. They may include increased publicity of the issue by electronic alerts and various media outlets, including the FDA (3) and CDC, as well as a cultural shift of increasing health compliance after the COVID-19 pandemic. Our preliminary results are encouraging and may indicate a shift in traditional practices of blood culture overutilization. A broader application of this diagnostic stewardship intervention has implications for cost-savings and effective utilization of collective resources.

## SUPPLEMENTAL MATERIAL

Supplemental material jcm.01701-24-s0001.docx EMR educational banner and hard stop alert.

ASM does not own the copyrights to Supplemental Material that may be linked to, or accessed through, an article. The authors have granted ASM a non-exclusive, world-wide license to publish the Supplemental Material files. Please contact the corresponding author directly for reuse.
