# Supplementary material for: Effective mitigation of blood culture bottle shortage with diagnostic stewardship interventions
Source: J Clin Microbiol. 2025 Jan 24;63(2):e01701-24. doi: 10.1128/jcm.01701-24 (PMC11837545; doi:10.1128/jcm.01701-24)
Supplement: Supplemental material — EMR educational banner and hard stop alert. [file jcm.01701-24-s0001.docx]

**Effective Mitigation of Blood Culture Bottle Shortage with Diagnostic Stewardship Interventions**

Jessica Hudson MD, MPH^1^, Guillermo Rodriguez Nava MD^3^, Mindy Marie Sampson MD^3^, Amy Chang MD^3^, Alex Maurice Dussaq MD, PhD^1^, Jorge Luis Salinas MD^3^, Angela Serbest BS^2^, Tho Pham MD^1^, and Niaz Banaei, MD^1,2,3^

^1^Department of Pathology, Stanford University School of Medicine, Stanford, CA

^2^Clinical Microbiology Laboratory, Stanford Health Care, CA, USA

^3^Division of Infectious Diseases & Geographic Medicine, Stanford University School of Medicine, Stanford, CA


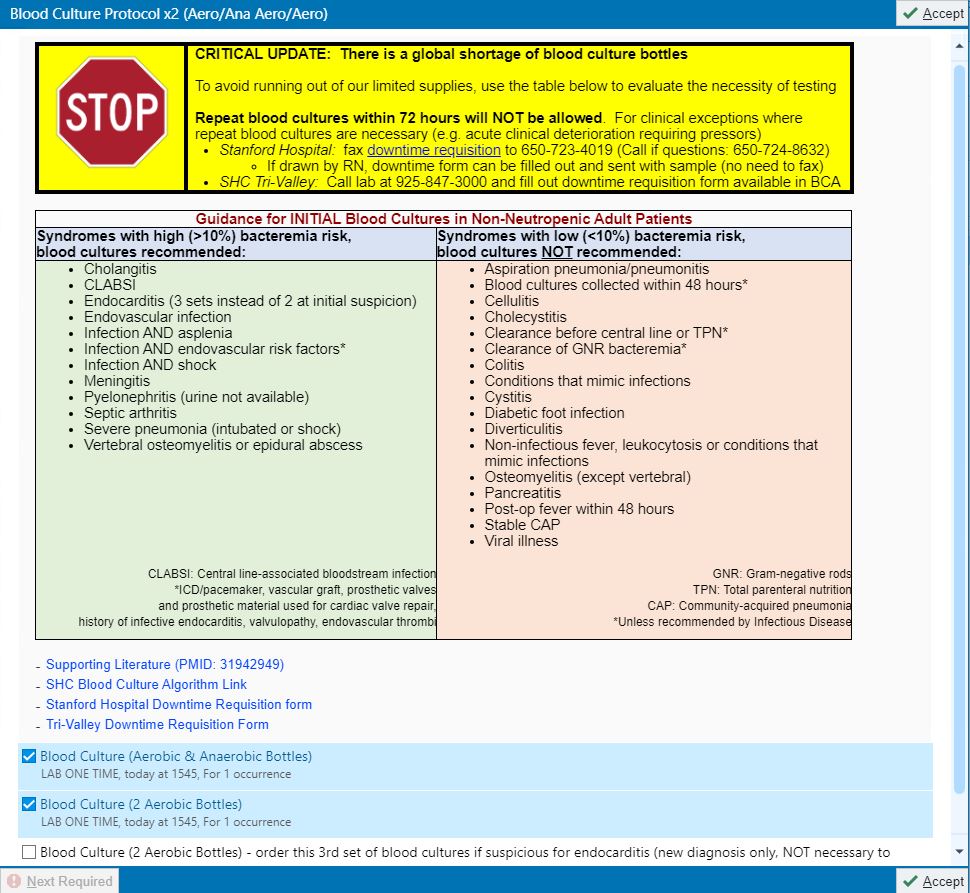


**Supplementary Figure 1. Epic alert to inform providers about the blood culture bottle shortage.** The alert includes an order panel with updated verbiage (highlighted in yellow) informing providers of the shortage. This was developed as part of the Johns Hopkins Prevention Epicenter Blood Culture Stewardship Collaborative. The content of the algorithm has been adapted from Fabre V, Sharara SL, Salinas AB, Carroll KC, Desai S, Cosgrove SE. Does This Patient Need Blood Cultures? A Scoping Review of Indications for Blood Cultures in Adult Nonneutropenic Inpatients. Clin Infect Dis. 2020, PMID: 31942949.


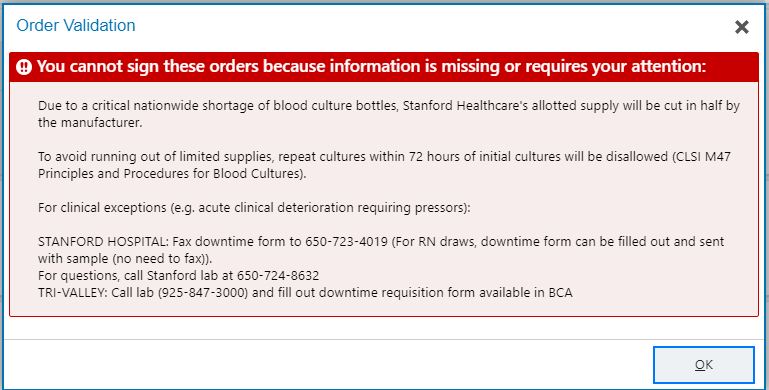


**Supplementary Figure 2. Hard stop alert appearing at time of blood culture order placement to block repeat blood cultures within 72 hours of baseline cultures.** A computer assisted algorithm was used to block repeat blood cultures within 72 hours of baseline cultures.
